# The New York State Board of Veterinary Medical Examiners

**Published:** 1897-03

**Authors:** 


THE New York State Board of Veterinary Medical Examiners deserves the thanks and approval of the veterinary profession in pushing to an issue the question of dehorning as a surgical operation coming within the provisions of the Act of the Empire State regulating the practice of the profession in that State. We have heard the argument advanced that the low prices received as remuneration for this work made it of little importance to the profession. This argument applies equally well to castration, ovariotomy, neurotomy, or any other everyday operation, and because it is done in a loose way or in a mechanical manner is no argument that it is not a surgical operation; and when so understood and performed it will receive a proper recompense.

